# Entrainment and motor emulation approaches to joint action: Alternatives or complementary approaches?

**DOI:** 10.3389/fnhum.2014.00754

**Published:** 2014-09-25

**Authors:** Lincoln J. Colling, Kellie Williamson

**Affiliations:** ^1^School of Psychology, Australian Catholic UniversityBrisbane, QLD, Australia; ^2^Department of Cognitive Science and Australian Research Council Centre of Excellence in Cognition and its Disorders, Macquarie UniversitySydney, NSW, Australia

**Keywords:** motor emulation, perception–action, entrainment, mechanistic explanation, joint action

## Abstract

Joint actions, such as music and dance, rely crucially on the ability of two, or more, agents to align their actions with great temporal precision. Within the literature that seeks to explain how this action alignment is possible, two broad approaches have appeared. The first, what we term the entrainment approach, has sought to explain these alignment phenomena in terms of the behavioral dynamics of the system of two agents. The second, what we term the emulator approach, has sought to explain these alignment phenomena in terms of mechanisms, such as forward and inverse models, that are implemented in the brain. They have often been pitched as alternative explanations of the same phenomena; however, we argue that this view is mistaken, because, as we show, these two approaches are engaged in distinct, and not mutually exclusive, explanatory tasks. While the entrainment approach seeks to uncover the general laws that govern behavior the emulator approach seeks to uncover mechanisms. We argue that is possible to do both and that the entrainment approach must pay greater attention to the mechanisms that support the behavioral dynamics of interest. In short, the entrainment approach must be transformed into a neuroentrainment approach by adopting a mechanistic view of explanation and by seeking mechanisms that are implemented in the brain.

## Introduction

The ability of people to engage in exquisitely coordinated action is a striking feature of collaborative endeavors, like music and dance. In these pursuits, people temporally and spatially align their actions with the actions of others, and failure to do so may be disruptive to the esthetic qualities of the performance. If one dancer was to fall behind, or rush ahead, such that they did not move in time with the other dancers it may lead to a breakdown in the performance. How is it that people are able to align their actions with such great precision? What are the processes that make this possible? And what is the best approach to begin to understand these phenomena? Ensemble music and dance performance are examples of the broader category of socially coordinated actions. Socially coordinated actions are any actions in which two or more agents are required to coordinate—for example, temporally align—their actions. At least two approaches have been adopted in building an understanding of how socially coordinated actions are achieved. The first, we will call the *entrainment approach*, which draws inspiration from the dynamics of coupled oscillators. The second, we will call the *emulator approach*, which views socially coordinated action as relying on predictive processes that allow agents to anticipate, and thus align with, the actions of their co-actors (Wilson and Knoblich, 2005; Colling et al., 2013a). The emulator approach tries to explain how the physical facts of the system—the system's parts, their organization, and their interactions—gives rise to the system's behavior—that is, it aims to provide *mechanistic explanations* (Craver, 2007; Bechtel, 2008). These two approaches are often conceived of as alternative or competing approaches to understanding a single process (Chemero, 2001; Kaplan and Bechtel, 2011; Schmidt et al., 2011, 2014; Stepp et al., 2011). For example, Schmidt et al. (2014) state that research on social entrainment that uses the tools of nonlinear dynamical systems (what we term the entrainment approach) demonstrate that there is no need for emulator mechanisms to explain this phenomenon. However, as we will show, the two approaches have distinct explanatory aims and seek to do their explaining in different, but not mutually exclusive, ways. Thus, rather than being viewed as alternatives, the two approaches should be viewed as complementary, with each approach playing a distinct role in the larger aim of understanding socially coordinated action.

The language of *entrainment*, a term that originates in physics, has recently been introduced into the psychological literature. Entrainment has been used to describe a wide variety of phenomena that involve two interacting systems that, over time, approach each other in the similarity of their behavior. For example, entrainment has been used to describe the tendency for two interacting individuals to approach the same emotional or affective state over the course of their interaction. It has also been used to describe the tendency for the movements of two individuals' limbs to adopt the same period and/or phase during coordinated rhythmic movements (see Phillips-Silver and Keller, 2012 for a review of entrainment phenomena). While this is quite a diverse range of phenomena, what they each have in common is a coming together and aligning of the behavior or states of two interacting systems that occurs as a consequence of their interaction.

One of the earliest scientific observations of entrainment phenomena was that two interacting, or *coupled*, oscillators will tend to adjust their behavior over time so that they become synchronized in phase and/or period. The first recorded observation of these phenomena came from Huygens, who observed that pendulum clocks, when placed side-by-side on a wall, tended to synchronize in period (Bennett et al., 2002). Furthermore, if the pendulums initially exhibited an 180° phase relationship they would tend to return to this phase relationship if one of the pendulums was perturbed. What these observations show is that when two oscillators (pendulums in the clocks) are coupled (placed together on the same wall) the movements of these two oscillators become *entrained* and they tend to be *attracted* toward particular stable patterns of coordination (talk of *attractors* will feature heavily in explanations offered by the entrainment framework outlined below). For example, Huygens's clocks that initially exhibited an 180° phase relationship were attracted toward this pattern of coordination such that they would return to this pattern when perturbed.

While Huygens's observations of entrainment were made in a purely artificial, mechanical system, many natural systems also seem to behave like coupled oscillators. For example, entrainment behavior has been observed in interacting fireflies flashing their lights (e.g., Buck, 1988), in rhythmic limb movements (e.g., Kelso, 1984; Mechsner et al., 2001), in music and dance (e.g., Large, 2000), and in other forms of socially coordinated actions, where two individuals attempt to coordinate their actions (e.g., Schmidt et al., 1990, 2011; Marsh et al., 2009). With such a wide variety of phenomena subsumed under the term entrainment, it does raise a question of what value the term might have. What can we gain from using the term entrainment so widely? For instance, might it be possible to gain some explanatory purchase on one entrainment phenomenon by using the same explanatory tools of similar entrainment phenomena? This has been the approach adopted by those working on the behavioral dynamics of coordinated actions, such as rhythmic inter-limb coordination (e.g., Kelso, 1984) and socially coordinated actions (e.g., Schmidt et al., 2011). This approach involves taking the explanatory tools that have been applied to the entrainment of simple coupled oscillators and applying them to coordination in more complex systems like rhythmic limb movements.

In this paper, we argue that the entrainment approach and the emulator approach have distinct, but compatible aims. We will make this argument by way of examples from the empirical literature. In this case, the examples from the empirical literature serve to illustrate the two styles of explanation. Importantly, our argument does not turn on the specifics of the explanada or the specifics of the data presented; rather, the reader is encouraged to pay attention to the style of explanation offered by each approach. With this in mind, we begin by outlining the entrainment approach and examine how it has been applied to coordinated behavior. Following this, we outline some key findings within the entrainment approach to demonstrate the *nature* of the explanations on offer, namely, general behavioral laws. We argue that this type of explanation leaves certain explanatory gaps in the *causal* story of the system's behavior. We then introduce the emulator approach, and highlight the different *kind* of explanation offered by this approach. In particular, we show how the emulator framework is built on certain physical facts about the brain, the kind of physical facts that do not enter into explanations offered by the entrainment approach. Doing so, we hope to demonstrate that explanations that only offer predictions of the regularities of a system at a behavioral level leave key questions unanswered, such as why these regularities exist and what the neural mechanisms are that give rise to them. In short, what is needed is a move away from exclusively studying entrainment at the behavioral level and a move toward what we term the “*neuroentrainment”* approach. That is, we argue for an approach to explaining entrainment that adopts the explanatory style of the emulator approach, but not necessarily the explanations of the emulator approach. This is not to discount behavioral work conducted within the entrainment approach. This work is important for constraining the general kind of mechanisms that underlie the phenomena of interest—that is, mechanisms that are capable of producing the observed behavioral dynamics. Viewed this way, entrainment and motor emulation are not alternative explanations; rather they can work to guide each other in developing multilevel—brain to behavior—explanations.

## Entrainment explanations and general laws

In his work with pendulum clocks, Huygens attempted to provide an explanation for the behavior of the clocks in purely physical terms. He explained the tendency for two pendulums to become synchronized in terms of being *caused* by vibrations sent along the wall. This causal explanation could, for example, explain why two clocks on the same wall would tend to become synchronized while two clocks on different walls would not, and why two clocks placed inline with each other would synchronize while two clocks placed perpendicular to each other would not. This type of explanation makes reference to the physical parts of the system—the pendulums and the wall—the organization of these parts—the placement of the clocks relative to the wall—and the operations performed by the parts—the transmission of the vibrations sent along the wall. The aim of this explanation is not to formulate precise predictions about how the dynamics of the system unfold over time; indeed, it might be difficult to formulate such predictions on the basis of this kind of explanation. For Huygens to make such predictions he would have needed the mathematics of differential equations, and Newtown's work that led to the development of these mathematical tools had only just begun (Bennett et al., 2002). Had Huygens been armed with these mathematical tools then it would have been possible for him to abstract away from the physical facts of the system and provide a formal model of the general laws that govern the dynamics of coupled oscillators. The apparent tension between these two tasks—providing a causal explanation that makes reference to the interacting parts of a physical system and uncovering the general laws that capture the behavior of a system—will be a major theme of this paper. It is this second approach, abstracting away from the physical facts of the system and attempting to uncover the general laws that capture the dynamics of the system, that has been adopted by those working within the entrainment approach. In the section that follows we outline how the entrainment approach has gone beyond pendulum clocks to formally modeling the dynamics of socially coordinated actions.

### Key developments for the entrainment approach

Some of the earliest experimental work within the entrainment approach was reported by Kelso (1984). The basic design of these experiments involved participants extending their forearms out in front of themselves with their wrists parallel to the ground and rotating their wrists in the horizontal plane. Participants were asked to begin cycling their hands slowly in an asymmetric mode (with one hand facing down while the other faced up) while slowly increasing the speed of rotation. When cycling speed reached a critical point there was a rapid breakdown of the coordination, with coordination rapidly being re-established in a symmetric mode. That is, once cycling speed reached a critical point there was a phase-transition from an anti-phase mode of coordination to an in-phase mode.

**Figure 1:**
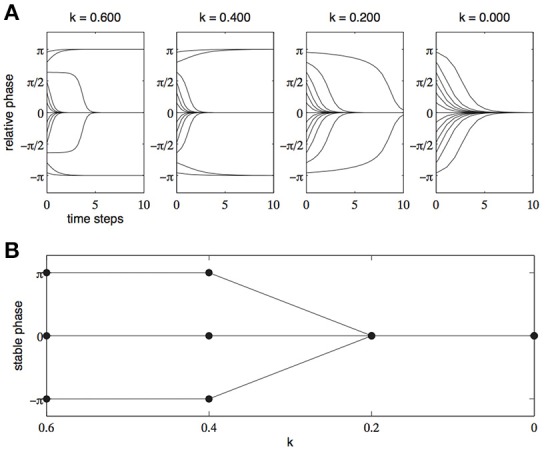
**(A)** Relative phase as a function of time for various initial phase relationships. High values of *k* represent slow cycling speeds and low values of *k* represent fast cycling speeds. **(B)** Stable patterns of coordination (attractors) as a function of decreasing values of *k* (increasing cycling speeds).

Haken et al. (1985) were able to formalize this finding using the mathematics of dynamical systems and the tools of dynamical modeling. The model developed by Haken et al.—the HKB model—was able to capture all the observed dynamics of the system of two hands by using only two simple parameters—cycling speed (1/*k*) and the relative phase (Φ) of the two effectors. The equation they devised exhibited all the characteristics of the coordinated limbs described by Kelso (1984). In particular, when the parameter *k* was set at relatively high values—which correspond to slow cycling speeds—two stable patterns of coordination emerged, an in-phase pattern (Φ = 0) and an anti-phase pattern (Φ = π). When the parameter *k* was set to relatively low values—which correspond to higher cycling speeds—only one coordination pattern was stable, the in-phase pattern (Φ = 0). The change in relative phase across time, for various cycling speeds, can be seen in Figure 1A while Figure 1B shows the stable modes of coordination for increasing cycling speeds (decreasing values of *k*)^1^.

For our present purposes, what is important here are not the specifics of the HKB model itself, but the general form of the explanation it provides. Once the dynamics of the system have been described (whether they have been formally modeled or whether the attractors have merely been identified) it is possible to abstract away from the physical facts of the system and explain the tendency of the system to settle into particular stable patterns of coordination in terms of the specific *attractors* that are present in the dynamical description of the system. For example, in the HKB model, the system has attractors at in-phase and anti-phase relationships when cycling speed is low. Once cycling speed passes a particular threshold value the anti-phase attractor is destroyed and only the in-phase attractor remains (see Figure 1B)

Originally, it was proposed that the stability of in-phase coordination might be the result of a bias toward symmetry of co-activated homologous muscles due to interactions occurring in the motor cortices of the brain (e.g., Johnson et al., 1998). However, because entrainment is a common feature of systems of coupled oscillators, and because the HKB equations make no reference to the physical parts of the system, researchers have sought similar systems that might also exhibit HKB-like behavior despite consisting of different physical parts. One such example is described by Mechsner et al. (2001). They suggested that activation of homologous muscles might not be necessary for a bias toward the in-phase pattern of coordination to appear. To examine this, they independently manipulated motor symmetry and perceptual symmetry by asking participants to perform a finger-wagging task with either both hands oriented the same way or with one palm-up and the other palm-down. Participants moved their index fingers side-to-side (in a wagging motion) either visually in-phase/anti-phase or motorically in-phase/anti-phase (motorically in-phase refers to contracting the same muscles in both hands). The results showed that the movements of the two fingers tended to settle into a stable pattern of coordination when they were visually in-phase, even when they were not motorically symmetrical.

Taken together, the findings of Mechsner et al. (2001) and the earlier work of Kelso (1984) and Haken et al. (1985) suggest that when two oscillators (for example, oscillatory limb movements) are *coupled* there is a tendency for the two oscillators to fall into a stable pattern of coordination regardless of the physical parts of the system. Thus, two physically disparate systems can be captured using the same explanatory tools, namely those of entrainment.

One particularly remarkable case of a system that differs radically from the case of inter-limb coordination, and yet exhibits the same dynamics, is found in socially coordinated action. Schmidt et al. (1990) report a series of experiments in which they were able to demonstrate that two limbs belonging to two distinct individuals obey the same dynamical laws as two limbs belonging to a single individual, such as the cases described by Kelso (1984) and Mechsner et al. (2001). In their experiments, Schmidt et al. (1990) asked two participants to sit side-by-side and to swing the lower part of their outside leg from side-to-side in time with a metronome. Participants were then asked to coordinate their movements with those of their partner so that the two legs were either in the same part of the cycle at the same time (in-phase relationship) or at opposite places in the cycle (anti-phase). Consistent with the predictions of the HKB model, and the findings from inter-limb coordination, it was found that both in-phase coordination and anti-phase coordination were stable at low cycling rates but that anti-phase coordination grew unstable as cycling rates increased.

### Explanations of entrainment without physical facts

In the examples outlined above, physically disparate systems have been subsumed within a single explanatory framework. The tendency of the systems to settle into particular stable patterns of coordination can be *explained* by the presence of particular attractors at those phase relationships. In the case of bimanual inter-limb coordination and socially coordinated action the presence of attractors at these phase relationships can be explained by the fact that these systems are governed by a general law, as described by the HKB equations. The same style of *explanation* can also be proffered for Huygens's clocks. However, this explanation is of a kind quite different to that which Huygens originally developed. Huygens offered an explanation in physical terms that made reference to the clocks sending vibrations through the wall (Bennett et al., 2002). These physical facts about the system—that the clocks vibrate and that these vibrations are transmitted by the wall—are absent in an explanation invoking the presence of particular attractors. While Huygens's explanation may lack the predictive power of a formal model derived from the mathematics of coupled oscillators, it does provide an explanation about why, for example, clocks on the same wall become entrained while clocks on different walls do not. The HKB equation makes no reference to the physical facts of the systems it describes—indeed it applies to physically very different systems—but it is able to predict the behavior of these systems very accurately. What the HKB equations fail to do is to provide a reason why these systems should even behave like a system of coupled oscillators that exhibit HKB dynamics in the first place. By analogy to Huygens explanation, what is serving as the wall through which vibrations can be sent? How do the interacting parts of the system give rise to this behavior? Some have argued that models like the HKB preclude breaking the system down into its component parts (Chemero, 2001). This is because there are no parameters in the equations that map onto parts of the system. Furthermore, because of the non-linear nature of the system it is not possible to break the behavior of the system down into component equations that can simply be added together. The two kinds of explanations, the dynamical model, of which the HKB is one example, and an explanation of the style offered by Huygens seem to be answering two different questions.

While the entrainment approach has attempted to abstract away from the physical facts of the system in favor of finding the general laws that govern the behavior of the system, the emulator approach has attempted to make sense of a system's behavior by examining how the physical features of the system give rise to the behavior. It is this approach that we turn our attention to in the next section.

## The emulator approach and mechanistic explanation

Where the entrainment approach to understanding socially coordinated action has attempted to abstract away from the physical facts by modeling coordination dynamics as coupled oscillators, the emulator approach has instead attempted to understand socially coordinated action by examining the mechanism that gives rise to the phenomenon. In particular, the emulator approach has sought to explain socially coordinated action in terms of inverse and forward models (Wolpert et al., 2003; Wilson and Knoblich, 2005; Csibra, 2008; Keller, 2012; Colling et al., 2013a). Many different formulations of the emulator approach exist, and each approach may differ in terms of the specific details. However, nothing in our argument turns on these details and, therefore, we outline something like a generic account drawing on specific details outlined in Colling et al. (2013a) and Wilson and Knoblich (2005) only when necessary. To show how it is the case that the emulator approach proposes mechanistic explanations, this section will first give an outline of the basic processes of inverse and forward models, which will be key to understanding the function of emulators. Following this, we will explain some of the functions that emulators perform, and we will show how these processes might be implemented in the brain. Once we have outlined the basics of emulators and some of their neural implementation, we will show how, by proposing that such a mechanism supports socially coordinated action, it is possible to make a number of predictions about the behavioral effects that should be observable in different experimental contexts.

**Figure 2:**
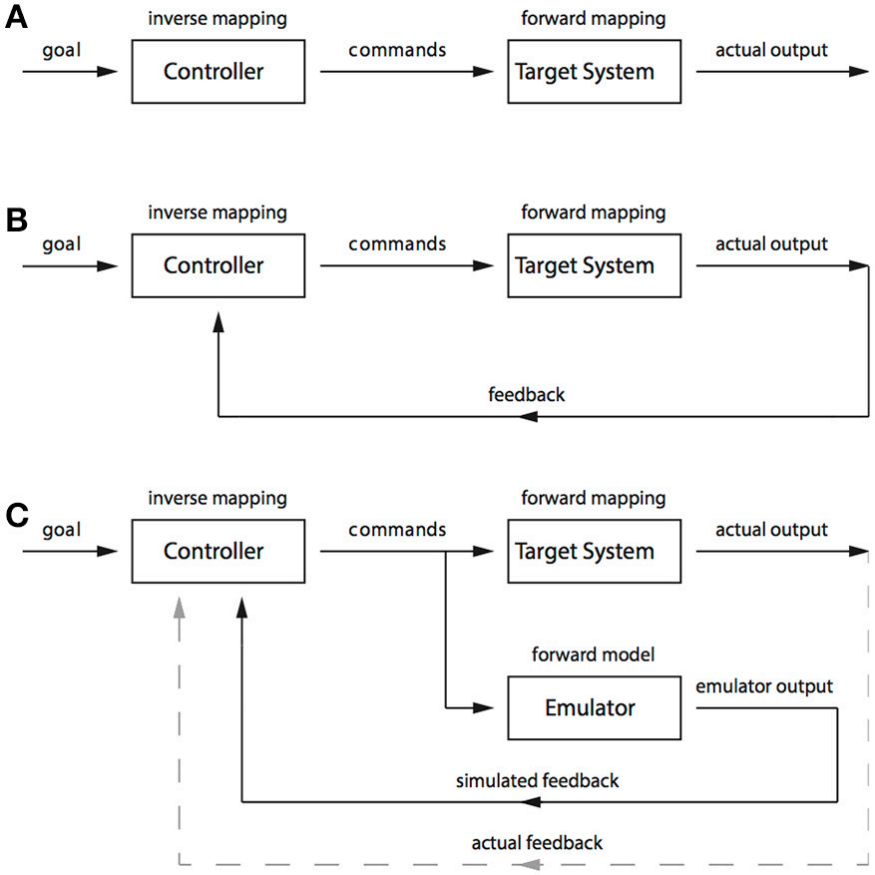
**(A)** A schematic of open loop control. **(B)** A schematic of closed-loop control. **(C)** A schematic of pseudo-closed-loop control.

Inverse and forward models are terms borrowed from control theory to describe mechanisms that can be used to control and predict the behavior of a system (for an introduction to control theory, see Golnaraghi, 2010). Inverse models are so named because they perform an *inverse* mapping from a desired state (or output) to the control commands necessary to produce that output. In a simple example of a room heater, a goal state might take the form of a particular room temperature. The control commands would be the power sent to the heating element, and they could be specified by an inverse model that maps room temperature to power level; for example, a simple control knob linked to a variable resistor where high resistance corresponds to lower temperatures and low resistance corresponds to high temperatures. While the controller, or inverse model, performs an inverse mapping, the target system (also called the *plant*) performs a *forward* mapping from control commands to the goal state (See Figure 2A). That is, it transforms the increases in power to increases in room temperature. Achieving the desired goal can be fairly difficult in such a system, because the appropriate control commands need to be specified exactly at the outset. If too much power is sent to the heating element then the room will get too hot and if not enough power is sent to the heating element then the room will not reach the desired temperature. To avoid having to specify the control commands precisely at the outset it is possible to include a sensor that measures the room temperature and feeds this information back to the controller. This *feedback* can be used to shut off the heating element when the room is getting too hot and to increase power to the element when the room is too cold (See Figure 2B).

In our simple example, it is clear that a control system using feedback (also known as a *closed-loop* control system, in contrast to an *open-loop* system that does not use feedback; see Grush, 1997) would be fairly successful at performing the task of achieving and then maintaining a desired goal. However, the system breaks down when accurate information can no longer be received from the sensors. At least two types of sensor problems can arise. First, information from the sensors might not actually reflect the true state of the room temperature because of a temporal lag that means the information sent back from the sensors instead reflects the temperature of the room as it was some time in the very recent past. Or second, the sensor readings may be intrinsically inaccurate because of, for example, sensor noise. Both of these problems can be overcome by using a forward model.

A forward model performs the reverse mapping of an inverse model and instead maps control commands to goal states. The forward model acts as an emulator of the plant by replicating its input–output relations. The forward model can be used to overcome problems associated with inaccurate sensor readings because information from the forward model can be substituted, entirely or in part, for feedback from the sensors. When a control command is issued to the plant a copy can be sent to the forward model (See Figure 2C). If the forward model is accurate then its output should track the output of the plant. In our room heater example, the room temperature increase produced by the room heater would be tracked by a simulated room temperature increase, and simulated sensor readings, produced by the forward model. In the case of sensor noise, these simulated sensor readings can be combined with actual sensor readings to produce a more accurate estimate of the actual room temperature. And in the case of delayed sensor readings, the simulated sensor readings can be wholly substituted for the actual sensor readings. The actual sensor readings can still be used as a training signal to ensure that the simulated sensor readings accurately track the actual sensor readings, but they no longer need to be used for control.

A key aspect of an emulator is that the plant can be taken off-line so that it does not produce any actual output. For example, the control commands, one copy of which is usually sent to the heating element while the other is sent to the forward model, can be sent to the forward model alone. In this way the system can be used to simulate^2^ a heated room and to simulate a change in temperature without producing any actual heating. Grush (1997) suggests that this fact makes emulators useful in cognitive systems because emulators make it possible for cognitive systems to engage in planning or to entertain counterfactuals by simulating or representing possible states of the world.

### Emulators in action control

Inverse and forward models—together known as *internal models*—are thought to play a vital role in action control (Wolpert et al., 1998). Inverse models act as controllers that are able to transform a desired limb trajectory into a series of motor commands necessary for producing that trajectory. The motor command and the desired trajectory are specified in different coordinate frames. The motor command specifies, for example, changes in the joint angles over time while the goal trajectory is specified in, for example, changes in visuo-spatial location over time. A key function of the inverse model is to transform or map trajectories in one coordinate frame into trajectories specified in another coordinate frame, just as the inverse model in the room-heating example—the control knob—mapped temperatures to resistance level.

Forward models, on the other hand, replicate the dynamics of, for example, the limb being controlled and, therefore, they can be used to predict how the limb will respond to the control commands issued by the inverse model. Because of sensory delays in obtaining feedback signals from the periphery, using a forward model to predict how the limb will move^3^ in response to particular control commands makes it possible to engage in planning without needing to specify the control commands precisely at the start of the movement (see, Wolpert and Kawato, 1998). Predictions from the forward model can also be used to estimate the state of the limb, by combining forward model predictions with sensory information (Wolpert, 1997). Running the forward model offline—that is, without producing motor output—can also be used to internally simulate limb movements; that is, to engage in motor imagery (Grush, 2004). And, as we will later show, internally simulating actions may also support social coordination of action.

How might inverse and forward models be implemented in the brain? While a complete treatment of the neural machinery that might support inverse and forward models—an area that is still undergoing active research—is outside the scope of this paper, it is possible to provide an overview of one aspect of these internal models that is, conceptually, quite straightforward (for a recent review of how internal models might be implemented in the brain the reader is directed to Ito, 2008). This is the possible implementation of coordination transformation in the cerebellum. One vital task performed by inverse and forward models is that they map trajectories in one coordinate space (for example, trajectories in visual space specified in spatial coordinates) onto trajectories in another coordinate space (for example, trajectories in a motor state-space). An early suggestion of how the brain might perform coordinate transformation was prompted by findings about the microarchitecture of the cerebellum (see Llinás, 1975 for an easy introduction to the cerebellum). Churchland (1989) outlines a simplified account of how the structure of the cerebellum might be able to perform coordinate transformation. Mathematically, to transform an n-dimensional input vector into an m-dimensional output vector it is necessary to multiply the input vector by an m × n dimensional transformation matrix. To do this, the first value of the m dimensional vector is multiplied by each of the n values in the first column of the m × n matrix. This column is then summed to yield the first value of the n dimensional vector. The process is then repeated for all the values of the input vector. In the example shown in Equation 1, a in the input vector is multiplied by *p*_1_ then *p*_2_, *p*_3_, and *p*_4_. These values are then summed to yield the value *x*. This process is then repeated to yield the value for *y*, and then for *z*.

$$\left[\begin{array}{cccc} a & b & c & d \end{array}\right]\;\cdot \;\left[\begin{array}{ccc} p_{1} & q_{1} & r_{1}\\ p_{2} & q_{2} & r_{2}\\ p_{3} & q_{3} & r_{3}\\ p_{4} & q_{4} & r_{4} \end{array}\right]\;=\;\left[\begin{array}{c} x\\ y\\ z \end{array}\right]$$

Equation 1. Matrix multiplication

**Figure 3:**
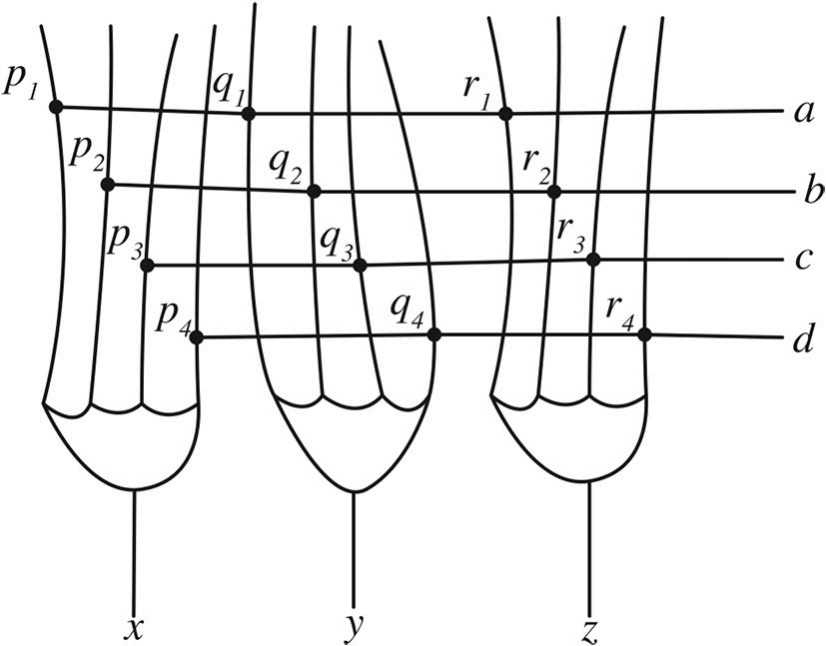
**A schematic of the cerebellum peforming matrix multiplication**.

The structure of the cerebellum may be ideally suited to performing this calculation. In the schematic in Figure 3, each of the four parallel fibers synapse onto the dendrites of three Purkinje cells. These synapses act as a transformation matrix where the summed activity from the four parallel fibers on one Purkinje cell determines the output value of that Purkinje cell. When this is repeated for all three Purkinje cells then this process transforms the three input values into four output values. The input and output values can be coded in terms of, for example, spiking rates, and the strength of the connection at the synapses can be used to specify the values of the transformation matrix. Since activity across the entire system happens in parallel this method performs the coordinate transformation rapidly enough for it to plausibly support coordination transformation in the action control system.

As with the discussion of the HKB model, what is important here are not the exact details of the mechanism proposed to underlie action control but rather the nature of the explanation offered by this account. For example, the coordination transformation mechanism may not be implemented in the cerebellum exactly as described above. And the details of what the forward model predicts—whether it predicts motor dynamics, sensory feedback, or both—are still open for empirical investigation (for example, Cisek, 2005). Rather, what is important is the general explanatory scheme whereby a function (motor control) is decomposed into component parts (inverse and forward models) and interactions between those parts—for example, feedback. These component parts can be broken down further into subparts and their interactions (coordinate transformation) and their neural implementation can be uncovered (in the microstructure of the cerebellum). As a result, the nature of the function is determined by the mechanism that implements it. The search for the mechanism is constrained by the nature of the function the mechanism is thought to implement. This is in contrast to the explanatory scheme favored by the entrainment approach where the task of explanation is to develop an account of the general laws that govern particular behaviors. Of course it is possible to do both (in the final section we will sketch how this might be possible), but it is the attempt to understand social motor coordination in terms of mechanisms that is the explanatory scheme favored by the motor emulation approach.

### Motor emulation and socially coordinated actions

According to the emulator framework, social motor coordination is supported by the same mechanisms that underlie action control (Wilson and Knoblich, 2005; Sebanz and Knoblich, 2009; Colling et al., 2013a). This proposal is partly based on the finding that overlapping neural networks are activated during action production and action observation (Gallese et al., 1996). In particular, premotor, parietal, and cerebellar regions are active both when observing actions and when performing actions (Molenberghs et al., 2012). Neurons in the superior temporal sulcus appear to be active during action observation only and they lack the motor properties of classical mirror neurons (Rizzolatti and Craighero, 2004; Molenberghs et al., 2012); therefore, these regions might be involved in the visual analysis of observed actions (Csibra, 2008). On the basis of the discovery that regions associated with action control are also activated during action observation, it has been suggested that these regions might play a role in prediction of *observed* actions, analogous to the role they may play during the performance of actions. In particular, it has been suggested that the predictive mechanisms (i.e., forward models), outlined above, that are involved in generating predictions during action performance may also be involved in generating predictions about actions during action observation (Wilson and Knoblich, 2005; Kilner et al., 2007; Colling et al., 2013a). Importantly, other proposals about the function of mirror neurons have been put forward (for example, Rizzolatti and Sinigaglia, 2010; Rizzolatti and Fogassi, 2014). However, our aim here is not to argue for a specific interpretation of mirror system function, but instead to illustrate how the emulator framework proposes mechanisms whose parts can be localized in the brain.

According to the general formulation of this account, visual analysis of the observed action, and conjectures about the co-acting agent's putative goals and intentions, are fed through the inverse model to generate a series of motor commands (Csibra, 2008; Jacob, 2008; Colling et al., 2013a). These motor commands can then be fed into a forward model to generate a real-time prediction about the unfolding action (Wilson and Knoblich, 2005; Colling et al., 2013a). That is, the mechanisms that support action control can be taken offline, and the observing agent can simulate the actions of their co-actor by using their action control system as an emulator.

Of particular interest for our current purposes are a series of experiments that have sought to understand what role forward models might play in socially coordinated action (Flach et al., 2003; Colling et al., 2013b, 2014). Several other findings including those involving self vs. other prediction (e.g., Knoblich and Flach, 2001; Knoblich et al., 2002), self vs. other coordination (Keller et al., 2007), self recognition (Flach et al., 2004; Repp and Keller, 2010; Sevdalis and Keller, 2010), and the role of self kinematic knowledge in visual (Daprati et al., 2007) and auditory (Repp and Keller, 2010) discrimination could be used to illustrate the kind of mechanistic explanation offered by the emulator account. However, we have chosen the examples because of the similarity in the paradigms used in these experiments and the experiments outlined in the entrainment section and because of the similarity of the phenomena being studied. These experiments have attempted to find evidence of a role for motor emulation in prediction of observed action by employing tasks that require participants to coordinate their action production with an observed action. These experiments are particularly relevant because the methods employed in these studies are superficially very similar to those employed in the entrainment approach. *The real-time prediction paradigm* employed in these experiments, as with the synchronization paradigm employed within the entrainment approach (e.g., Schmidt et al., 1990) requires participants to align a motor response with a motor response performed by a conspecific. Both the emulator approach and the entrainment approach try to *explain* coordination of action between individuals; however, as we shall see, what the *explanation* looks like in each approach is very different.

The logic of the real-time prediction paradigm is that if observers are to align their responses with the actions of an observed conspecific then they must first generate a prediction about when specific features in the movement will occur. (These are termed *critical points* and they usually occur when the conspecifics movement changes, for example, in direction from an upward movement to a downward movement). Further, if the observer uses their own internal models for generating these predictions then the accuracy of these predictions should be influenced by the kinematics of the observer's actions. That is, observed actions that have the same kinematic properties as the observer should be predicted more accurately, because the dynamics of the observer's forward model will more closely match the observed action. Furthermore, if motor emulation requires the observer to first map the observed actions onto their own action system, then predictions should also be influenced by the form of the observed actions. For example, if the observed actions contain information about the configuration of the effectors producing the movements then this should facilitate prediction relative to the case where this information is missing but all dynamic information remains. Note that these predicted effects flow directly from the hypothesized mechanism. The entrainment approach, which abstracts away from the mechanism, and instead tries to understand social motor coordination by uncovering the general laws that govern the dynamics of social motor coordination, makes no such prediction.

**Figure 4:**
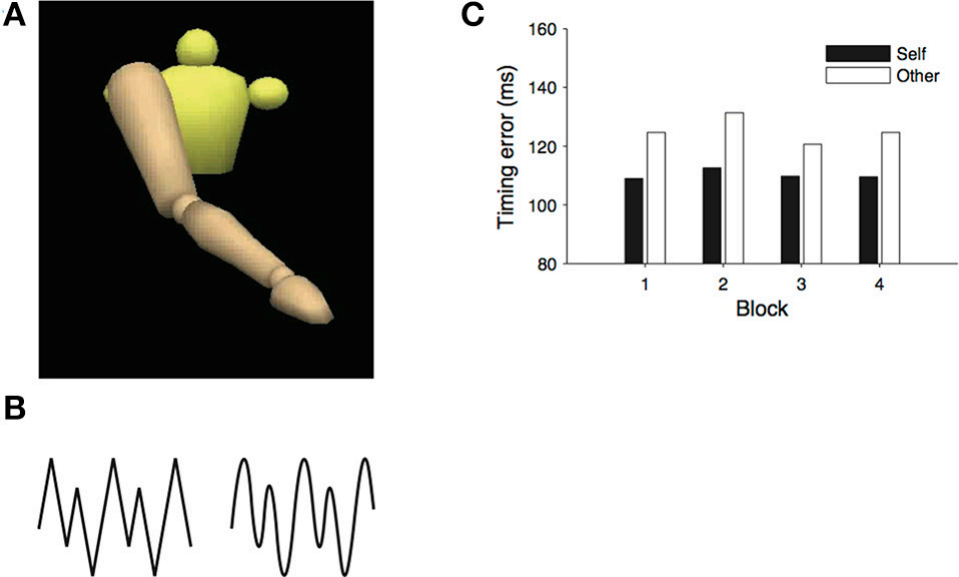
**(A)** The mannequin stimuli, **(B)** movement patterns, and **(C)** results from Colling et al. (2014).

The version of the real-time prediction paradigm employed by Colling et al. (2014) was split into two phases. In the first phase, participants were asked to perform a series of arm movements as if drawing zigzag or wave shapes on a blackboard, while their movements were recorded with motion capture. The peak heights in the patterns were irregular and alternated from large to small. In the second phase of the experiment, participants were asked to view the motion capture recordings, rendered as animated mannequins and to align a produced action (a button press response) with the critical points in the observed stimulus (see Figure 4,^4^). Each participant viewed movements that had been recorded during their own action performance as well as traces recorded from one other person. The timing error between the button press responses and the occurrence of the critical point in the stimulus can be used as a measure of prediction accuracy, or the accuracy of behavioral alignment between the observer and the observed action.

If observers use a forward model of their own kinematics to generate predictions about the observed actions—that is, if action prediction relies on a mechanism that is composed, in part, of a forward model—then alignment should be more accurate when observers view traces that they had themselves produced because the forward model would more accurately replicate the dynamics of these displays. Consistent with the predictions of the emulator hypothesis, timing error was lower when observers viewed recordings of their own actions. Subsequent control experiments were able to rule out the explanation that superior performance on the self-generated stimuli was simply the result of the idiosyncrasies of the timing of the participants' action production but was indeed dependent on the kinematic properties of the observed action.

Importantly, not all cases where observers view recordings of their own actions produce a prediction advantage for self-generated actions. However, these failures are informative because they are expected when viewed in light of the mechanism thought to produce the self-prediction advantage. That is, given a description of the mechanism thought to underlie the effect it is possible to give a causal story about why some systems will behave a certain way and why others will not. By analogy to Huygens's observations, given his explanation based on the transfer of vibrations through the wall it is possible to explain why clocks on the same wall become entrained and why clocks on different walls do not. This is in contrast to the entrainment approach, which seeks to uncover general laws governing the dynamics of a system, where it is not possible to give a causal story of why these laws should apply to some systems and not others.

The results that demonstrate this distinction are a series of observations by Flach et al. (2003) where they found that the self-prediction advantage was eliminated when the observed actions were produced by performing waves and zigzags with regular (that is, not alternating) peak heights. The finding that the kinematic properties of the traces (whether they were derived from self-produced or other-produced action) only made a difference when the traces were not uniform is to be expected, because the task can be performed in a qualitatively different way depending on whether the peaks are regular or irregular. For the uniform traces the stimulus is isochronous with the time between each of the critical points being roughly equal; therefore, the movement time required to produce the first upstroke provides all the information needed to predict the timing of all the subsequent critical points. That is, when the peaks are regular, it is not necessary for observers to engage in action prediction and instead they can perform the task solely on the basis of the timing information present in the first upstroke.

A second claim of the emulator hypothesis is that action prediction relies on a mechanism that is composed, in part, of a system that transforms the observed actions through visual analysis into a set of motor commands, which are then fed through the forward model. That is, the action prediction mechanism contains something like an inverse model. Therefore, disrupting the visual analysis process, by using stimuli that do not uniquely specify how the observed action was produced should disrupt the prediction process. To test this prediction, Colling et al. (2013b) employed two sets of stimuli. The first set of stimuli were once again mannequins performing irregular zigzag and wave movements. And the second set of stimuli consisted of only a single moving point that tracked the movement of the mannequin's hand. In the point stimuli, all the information that the observer required to align their actions with the observed action was present in the stimuli. However, the point stimuli contained no information about the configuration of the observed agent's limbs. That is, the observed trajectory was ambiguous as to the action that was actually used to produce the trajectory with any number or combination of hand, wrist, elbow, or shoulder movements capable of producing the trajectory—although the movements in both conditions were identical and generated from the same motion capture data.

The results of the experiment showed a clear advantage for aligning responses with the mannequin stimuli relative to the point stimuli. Importantly, this advantage was not simply due to low-level stimulus features (such as added complexity in the mannequin stimuli), because a second set of participants who had no experience with the observed stimuli did not show any differences in alignment accuracy between the stimuli. Subsequent analyses suggested that these naïve observers just produced button presses at a regular interval presumably having formed a representation of the temporal structure of the stimulus on the basis of timing information available in the first upstroke—a process that would not require engaging a motor emulator mechanism. From the examples outlined it should be clear that mechanistic explanations make it possible to give the kind of causal explanations that general laws do not permit. In particular, on a mechanistic account it is possible to say why particular systems exhibit the behavior they do on the basis of the structure of the system. And it is possible to say why particular effects occur in some circumstances and not others on the basis of the functioning of the system. For example, a mechanistic account, like the emulator approach, provides an explanation of why self–other differences emerged in the experiments of Colling et al. (2014) and why self–other differences are unlikely to emerge under other circumstances—for example, the Regular condition (uniform peak heights) of Flach et al. (2003; Experiment 1). And a mechanistic account is able to explain why Colling et al. (2013b) found that experienced observers were able to align their behavior better with a mannequin stimulus relative to a point stimulus.

## Neuroentrainment or using mechanistic explanations to explain entrainment phenomena

The emulator approach and the entrainment approach to explaining socially coordinated action have sometimes been viewed as alternatives. It is true that the type of explanation in each case is distinct. The entrainment approach explains by invoking explananda such as attractors and system dynamics while the emulator approach invokes explananda such as forward models and inverse models, which can be cashed out in terms of how they are realized in the brain. However, it would be a mistake to judge them to be alternative explanations of socially coordinated action, from which we must favor only one approach. From the preceding section it should now be clear that the entrainment approach and the emulator approach do not only invoke different explananda, they also provide different *kinds* of explanations. The entrainment approach proposes general laws and the emulator approach proposes mechanisms. We have argued in favor of proposing mechanisms, because this makes it possible to provide a kind of causal story that the general laws approach does not allow. By proposing general laws it is not possible to say why a particular system exhibits particular behavioral regularities and others do not. In contrast, a mechanistic explanation explains why certain behavioral regularities exist with reference to the system being composed of interacting parts organized in a particular way. In the case of the emulator approach, an attempt is made to explain behavioral phenomena by virtue of the neural parts and operations that give rise to them. Developing models that only describe behavioral regularities, as per the entrainment approach, cannot be the only way forward because these models will fail to explain what gives rise to these phenomena. We need to adopt the explanatory tools of the emulator approach, a *neuroentrainment* approach, that explains how the organized parts and operations of a mechanism gives rise to the behavioral regularities of interest.

Some initial steps have been taken to provide mechanistic explanations of why the effects observed in coordination experiments occur by explaining these effects in terms of brain activity (Jirsa et al., 1998; Jantzen et al., 2008; Tognoli and Kelso, 2014). Inline with the explanatory style of the emulator framework, what these approaches share is that they attempt to explain the observed behavioral dynamics in terms of the interacting parts of the system that produces the behavior—specifically, neural ensembles, which exhibit dynamics similar to those observed at a behavioral level (see Friston, 1997). For example, Jirsa et al. (1998) have built a model that explains the phase shifts observed at the behavioral level in terms of the dynamics of the underlying neural populations. Interestingly, work that has looked at the link between large-scale brain activity and coordination has implicated regions often associated with the emulator system, namely premotor and parietal regions that form part of the mirror system (Wilson and Knoblich, 2005; Colling et al., 2013a). For example, Jantzen et al. (2008) has looked at the link between large scale brain dynamics and unimanual coordination with an auditory signal. This work suggests that unstable coordination is associated with increased neural coupling between premotor and supplementary motor regions, suggesting an increased involvement of motor planning regions is needed to maintain coordination. Interestingly, these brain regions have also been implicated in action prediction, as well as prediction more generally (Schubotz, 2007), and may form part of the emulator system (Wilson and Knoblich, 2005; Csibra, 2008). Increased involvement of regions associated with motor emulation and motor planning may explain why action prediction is sensitive to observed kinematics when attempting to align behavioral responses with unstable stimuli such as irregular up and down arm movements (Colling et al., 2010, 2014) but not when aligning behavioral responses with stable, isochronous stimuli like regular wave and zigzag patterns (Flach et al., 2003; Experiment 1: regular pattern). This raises the possibility that the neural system that supports the effects observed in entrainment experiments might implement an emulator, and therefore these effects may be the result of the dynamics of the emulator. Indeed, Tognoli et al. (2007) has specifically tried to use the mechanisms proposed by emulator theory to explain entrainment phenomena. However, it should be noted that our argument does not turn on whether entrainment phenomena can be explained by motor emulation. Rather, a neuroentrainment approach should seek to explain entrainment phenomena using the explanatory style of the emulator approach—that is, mechanistically—and not necessarily by using the same mechanisms. What these mechanisms turn out to be is a question left for future empirical work.

In arguing in favor of the mechanistic explanation of the emulator approach we do not mean to discount the importance of uncovering behavioral regularities. Here, and elsewhere (e.g., Bechtel and McCauley, 1999; Bechtel, 2008; Colling and Roberts, 2010), it has been argued that making conjectures about the mechanism that underlies a phenomena of interest—for example, social motor coordination—should serve as a guide to predicting possible behavioral effects that the mechanism gives rise to. For instance, Bechtel and McCauley (1999) put forward the notion of a heuristic identity theory in which they argue that claims about the neural underpinnings of psychological phenomena should serve as a guide for future empirical research. This bottom-up route has been adopted by the emulator approach where findings about forward models and the mirror system led to the predictions that were tested using the real-time prediction paradigm. However, a top-down route, where the behavioral effects serve as a guide for uncovering mechanisms is an equally valid approach. The usefulness of this approach is clearly exemplified by the work of Jirsa et al. (1998). Indeed, the most fruitful approach might be to combine bottom-up and top-down approaches so that precise descriptions of the dynamics of phenomena and conjectures about the mechanisms that underlie these phenomena mutually constrain explanatory endeavors. Viewing the approaches as complementary will only improve our understanding of these phenomena.

Abandoning an exclusive focus of explanations based on general laws and adopting explanations based on mechanisms may also have deeper philosophical implications for the entrainment approach. Many researchers working on entrainment are staunchly anti-representationalist (Chemero, 2001; Schmidt et al., 2011; Stepp et al., 2011). While mechanistic explanations need not be representational explanations, they tend to be amenable to “representation hunting” (Zednik, 2011). The emulator approach is representational in that the forward model carries information about the musculosketal system and its operation is determined in virtue of carrying this information. Similarly, the search for mechanisms that underlie entrainment behavior, as seen in the work of Jirsa et al. (1998) and Jantzen et al. (2008), appears to make claims about a mechanism in which parts carry information and perform their function in virtue of the information they carry. Thus, such explanations may turn out to be representational. It is outside the scope of this paper to fully spell out the implications that a future neuroentrainment approach would have for the debates surrounding representational and non-representational views of cognition but it is a live area for further research.

## Conclusions

In this paper we have argued that the entrainment approach and emulator approach do not provide alternative explanations of socially coordinated action. Rather the two approaches offer distinct styles of explanation that cannot be viewed as competing explanations because they have distinct explanatory aims. To demonstrate this, we have shown by way of example how the entrainment approach offers explanations in the form of general laws that govern the behavioral dynamics of the systems under study. And we have shown by way of example how the emulator approach instead offers explanations in the form of mechanisms. The mechanistic explanation offered by the emulator approach makes claims about specific parts of the mechanism that can be localized to the brain (e.g., the cerebellum and mirror neuron system) and claims about the functions of these parts (e.g., that they function as internal models). Furthermore, we have argued that mechanistic explanations are superior because only they can provide the kind of causal story that explains why certain systems or tasks will exhibit certain behavioral features while other systems or tasks will not. As a result, we have argued that the explanations offered by the entrainment approach are not sufficient. To provide sufficient explanations of the explanatory targets discussed in the entrainment literature a mechanistic approach is needed. We have termed this new approach the *neuroentrainment* approach because any mechanistic explanation must ultimately seek to explain the observed behavioral dynamics in terms of parts and operations that can be localized in the brain.

### Conflict of interest statement

The authors declare that the research was conducted in the absence of any commercial or financial relationships that could be construed as a potential conflict of interest.

## Footnotes

^1^The Matlab code for generating these figures is available at http://research.colling.net.nz/code/hkb-demo

^2^Simulate is here used as a general term not to refer to a specific class of theories—for example, simulation theories (see Grush, 2004).

^3^Predictions about limb movements can also be fed into a second forward model that predicts the sensory feedback that will result from a particular movement (Wolpert, 1997).

^4^A video sample of the stimulus is available at http://research.colling.net.nz/stimuli/mannequin
